# Functional Assays to Guide Personalized Oncological Treatment of Patients with Soft-Tissue Sarcomas

**DOI:** 10.3390/cancers17213452

**Published:** 2025-10-28

**Authors:** Sude Yarar, Panagiotis Tsagkozis

**Affiliations:** 1 Istanbul Faculty of Medicine, Istanbul University, Istanbul 34452, Turkey; sudeyarar@ogr.iu.edu.tr; 2 Department of Molecular Medicine and Surgery, Karolinska Institutet, 171 76 Stockholm, Sweden; 3 Karolinska University Hospital, 171 64 Solna, Sweden

**Keywords:** soft-tissue sarcoma, chemotherapy, personalized treatment, functional assays

## Abstract

**Simple Summary:**

Soft-tissue sarcomas that have spread to the body are often treated with chemotherapy. Such treatment has limited efficacy. In order to improve outcomes, focus has been placed on personalized treatment, where each tumor is analyzed to identify drugs that are most likely to have an effect. This can be done based on information from the genetic material of the tumor cells or direct tests of the drugs on cells or pieces derived from the tumor mass. The latter, known as functional assays, have advantages since they allow for a direct analysis of the effect of each drug. Functional assays have been introduced in the management of soft-tissue sarcomas, and they are reviewed in the present manuscript. Although still at an early stage, this technology has shown promising results, which may improve patient outcomes.

**Abstract:**

**Background/Objectives**: Soft tissue sarcomas (STSs) are rare tumors arising from mesenchymal tissues, comprising over 100 distinct histological subtypes with varying biological behaviors, metastatic patterns, and treatment responses Despite advances in multimodal therapy, the overall survival of patients with metastatic STS is poor, mainly due to the weak response to conventional chemotherapy based on doxorubicin and ifosfamide. **Methods**: This review examines the evolution from traditional one-size-fits-all treatments to personalized medicine strategies, primarily focusing on assays based on patient-derived tumor samples, and it highlights their emerging role in guiding personalized treatment decisions and improving clinical outcomes in STS. These approaches, also known as functional precision oncology, are a step closer to the clinical situation as compared to other personalized therapies that rely on the identification of targetable genomic alterations using high-throughput technologies such as whole-genome sequencing, which have thus far failed to show convincing responses in STS treatment. **Results**: The main functional precision oncology platforms tested in patients with STS are in vitro cell viability tests, organoid cultures, and patient-derived xenografts. Each has advantages and limitations. In this context, in vitro drug sensitivity using cell suspension or organoids has shown a strong correlation with clinical responses. Furthermore, organoids matched the original tumor histology and microenvironment to a satisfactory degree. Establishment of xenografts proved feasible in the majority of patients; the technique could also preserve the tumor architecture and displayed high physiological relevance to the clinical situation. **Conclusions**: Although a major clinical study directly comparing conventional chemotherapy to personalized treatment guided by functional assays is yet to be published, this approach has gained popularity given the low efficacy of personalized medicine based on genetic alterations. The results thus far show promise for a better outcome for patients with metastatic STS.

## 1. Biology and General Principles of Soft Tissue Sarcomas

Soft tissue sarcomas (STSs) are a diverse group of malignant tumors originating from mesenchymal tissues such as muscle, fat, nerves and connective tissue [1]. Although they represent less than 1% of all adult cancers, STSs are characterized by significant heterogeneity in their histological subtypes and biological behavior, which complicates diagnosis and treatment [2]. The tumor biology of STS is complex and involves diverse genetic and molecular alterations that influence tumor growth, invasion, and response to therapy [3]. This heterogeneity underscores the need for accurate classification and individualized therapeutic strategies. 

STSs primarily spread through the bloodstream, most commonly to the lungs, which are the leading site of metastasis [4]. Although less frequent, certain subtypes such as angiosarcoma and epithelioid sarcoma may also metastasize via the lymphatic system [5,6]. In addition to distant metastases, many STSs aggressively invade surrounding tissues where they first arise, making surgical removal more complex and increasing the risk of local recurrence [7,8].

Surgery remains the mainstay of treatment for localized STS [9,10]. The goal is complete resection with negative margins to reduce the risk of local recurrence. Radiotherapy is often used as an adjuvant treatment [11,12,13]. It may be administered preoperatively to shrink the tumor and facilitate resection or postoperatively to eradicate residual tumor cells. Radiotherapy is particularly beneficial for large, high-grade tumors or those located in anatomically complex areas [14].

The clinical behavior of STS varies widely depending on subtype and grade [15]. Some tumors grow slowly and remain localized for years, whereas others are aggressive from the outset. Understanding these growth patterns and the molecular mechanisms underlying them is essential for improving patient outcomes. As research advances, there is growing recognition that personalized treatment approaches tailored to each tumor’s unique biology may be key to achieving better clinical results [16,17].

### Conventional Chemotherapy

Chemotherapy is an important treatment for STS when the tumor is at high risk of relapsing after surgery or has already metastasized [18,19]. However, in slow-growing or localized tumors, chemotherapy is less commonly used, and its clinical benefit remains uncertain. 

The standard first-line chemotherapy for many types of advanced STS involves anthracycline-based regimens, most commonly doxorubicin, either alone or in combination with ifosfamide [20,21,22]. Although combination therapy may provide higher response rates, it is also associated with increased toxicity and has not consistently demonstrated a significant overall survival advantage compared to monotherapy [18]. After progression on first-line therapy, several second-line agents are available, including gemcitabine, docetaxel, trabectedin, eribulin, pazopanib, dacarbazine, and ifosfamide. These drugs show varying degrees of activity depending on the histological subtype. For example, trabectedin demonstrates particular efficacy in translocation-associated sarcomas such as myxoid liposarcoma, while pazopanib, a multi-targeted tyrosine kinase inhibitor, is approved for non-adipocytic subtypes including leiomyosarcoma and synovial sarcoma [16,23,24] (Table 1).

Despite the availability of multiple chemotherapeutic options, their overall effectiveness remains limited due to the biological heterogeneity of STS. Response rates vary widely among patients, and treatment tolerance can be challenging. Conventional chemotherapy is still largely based on histological subtype rather than predictive biomarkers, and treatment selection often depends on clinical judgment rather than tumor biology. These limitations highlight the need to move beyond traditional cytotoxic regimens and develop personalized treatment strategies that incorporate molecular and functional characteristics to improve therapeutic outcomes [25].

## 2. Personalized Treatment Assays for Cancer Patients

### 2.1. DNA-Based Assays

Recent advances in genomic medicine have enabled the widespread use of DNA-based assays in cancer diagnostics and treatment planning. Techniques such as targeted gene panels and next-generation sequencing (NGS), including whole-genome and whole-exome sequencing, have significantly contributed to the identification of oncogenic mutations, gene fusions, copy number variations, and other alterations that drive tumorigenesis [26,27]. This molecular information has supported the development of targeted therapies in several malignancies, such as EGFR inhibitors in lung cancer and BRAF inhibitors in melanoma. 

In soft tissue sarcomas (STSs), the use of DNA-based assays has evolved over time, beginning with PCR techniques for detecting specific gene fusions, followed by multi-gene panels, and more recently comprehensive NGS technologies. These approaches have improved diagnostic accuracy and enabled deeper molecular profiling; however, their impact on treatment decisions remains limited due to the rarity of actionable mutations in most STS subtypes [28]. While specific translocations such as SS18-SSX in synovial sarcoma or FUS-DDIT3 in myxoid liposarcoma assist in precise subtype classification, they often lack direct therapeutic implications. 

Despite their transformative role in oncology, DNA-based assays have important limitations. Many detected genetic alterations are not “actionable,” meaning there is no effective targeted therapy available. Additionally, the presence of a mutation does not always correlate with drug sensitivity due to factors such as post-transcriptional regulation, epigenetic modifications, and the influence of the tumor microenvironment. Tumors also exhibit clonal heterogeneity and can evolve dynamically under therapeutic pressure, which static genomic data may fail to capture [29,30,31]. Consequently, while genomic profiling is essential for diagnosis and research, it frequently lacks the functional context needed to predict real-time therapeutic response. 

The application of DNA-based assays in STS has proved particularly complex due to their rarity and extreme heterogeneity [32]. Outside gastrointestinal stromal tumors (GIST), where KIT and PDGFRA mutations predict response to imatinib, actionable genomic alterations are uncommon in most STS subtypes [33]. Moreover, tumors harboring similar genomic changes may still differ significantly in growth rate, metastatic potential, and treatment sensitivity, indicating that non-genetic factors play a major role in STS progression and therapy resistance. 

Therefore, although DNA-based profiling remains fundamental for diagnosis and molecular classification, its ability to guide effective treatment in most STS subtypes is still limited. These limitations have led to growing interest in functional assays, which directly measure tumor behavior and drug sensitivity using patient-derived material. Functional approaches can validate genomic findings or provide actionable insights when DNA-based testing falls short, making them a promising complement to purely molecular strategies [34].

### 2.2. Functional Assays

Functional assays offer a direct evaluation of tumor response to various drugs using live tumor cells or tissues obtained from patients. By assessing drug sensitivity in real time, these platforms aim to better simulate the in vivo environment and capture the complex interplay between genetic, epigenetic, and microenvironmental factors that influence treatment outcomes [35]. Several categories of functional assays have been explored in cancer research:

- 2D Cell Viability Assays (e.g., MTT, ATP-luminescence): Widely used for drug screening in single-cell suspensions, but they lack three-dimensional structure and microenvironmental fidelity [36].
- 3D Organoid Cultures: Derived from patient tumors, organoids preserve the tumor’s histology, architecture, and some degree of microenvironmental complexity. They have shown strong correlation with clinical responses and are more predictive than traditional 2D cultures [37,38,39].
- Patient-Derived Xenografts (PDX): Involve implantation of human tumor tissues into immunocompromised mice. These models maintain tumor-stroma interactions and architecture, and have been highly predictive of drug efficacy. However, their use is limited by high cost, long development time, and low scalability [40,41].
- Ex Vivo Tumor Slices: Maintain native cell–cell interactions and can be used for short-term drug exposure studies. Although promising, data on their routine clinical application remain limited [42,43,44].
- Microfluidic Platforms (“tumor-on-a-chip”): Offer dynamic culture conditions that better mimic physiological flow and intercellular interactions. These platforms are innovative but remain largely experimental [45,46,47].
- High-Content Imaging and Live-Cell Assays: Allow for real-time monitoring of drug effects on cell morphology, apoptosis, and cell cycle, providing mechanistic insights beyond simple viability metrics [48,49].

Among these platforms, organoid and PDX models are the most widely studied in the context of STS. Notably, STS organoids have shown strong agreement between ex vivo drug sensitivity and patient responses, indicating that they may help guide personalized therapy. PDX models, while slower to develop, remain a gold standard for preclinical validation due to their high physiological relevance. However, both models face practical limitations, and their integration into routine clinical workflows is still challenging. Other approaches such as tumor slice cultures and microfluidic models represent emerging technologies, but studies specifically focused on STS remain limited. 

## 3. Functional Assays in Soft-Tissue Sarcomas

### 3.1. Cell Line-Based Assays

Cell line-based assays represent one of the earliest and most widely adopted functional platforms for evaluating drug sensitivity in cancer research. In the context of STSs, which are rare and biologically heterogeneous, these assays have gained renewed interest as a practical tool for individualized drug response profiling. They are considered more accurate than the use of commercially available STS cell lines, the most common of which are presented in Table 2. The general approach involves isolating tumor cells directly from patient-derived samples, propagating them in vitro, and systematically exposing them to a panel of chemotherapeutic or targeted agents. Drug efficacy is then evaluated based on quantitative changes in cell viability or proliferation, often through standardized readouts such as ATP-luminescence or MTT test [50,51,52,53].

**Figure 1:**
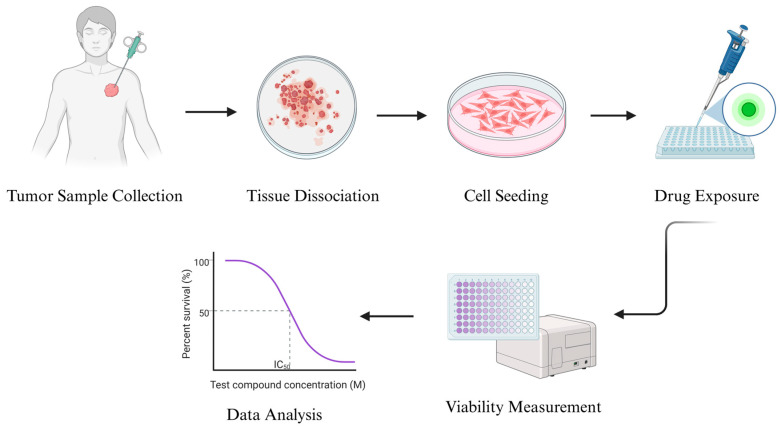
Establishment of soft-tissue sarcoma cell lines and in vitro analysis of drug efficacy. Created in BioRender.com.

The technical workflow typically begins with surgical or biopsy-derived tumor tissue, which is enzymatically digested to yield a single-cell suspension. Cells are seeded in two-dimensional (2D) culture formats, where they adhere and proliferate under optimized conditions. Once a stable monolayer is established, drug exposure is initiated across a range of concentrations. After 48–72 h of incubation, endpoint assays are performed to evaluate drug effects on tumor cell viability or survival. This process yields dose–response curves and IC50 values, which serve as surrogate markers of clinical sensitivity or resistance [54] (Figure 1).

Recent studies have demonstrated that patient-derived cell assays can produce results that correlate with clinical responses in STS. In vitro resistance patterns to agents such as doxorubicin and ifosfamide have shown significant concordance with patient outcomes. Furthermore, stratification of patients based on these assay results has been associated with statistically significant differences in recurrence-free survival, highlighting the potential of 2D assay platforms to provide clinically actionable information [55].

In addition to retrospective validation, some studies have also applied this methodology prospectively, using in vitro drug sensitivity results to guide clinical decision-making. In these cases, treatment regimens selected based on cell assay data have led to measurable clinical benefit, including partial responses and prolonged disease control in patients with otherwise chemoresistant disease [56]. Furthermore, the method has been successfully applied to rare histological subtypes such as undifferentiated pleomorphic sarcoma, synovial sarcoma, and leiomyosarcoma, suggesting that it may help tailor therapy even when genomic targets are limited. 

Despite certain limitations—such as the lack of tumor architecture, immune components, or stromal interactions—cell line-based assays remain advantageous due to their rapid turnaround, cost-efficiency, and scalability. These properties make them suitable as a first-tier functional screening tool, especially when fresh tumor tissue is limited or when quick treatment decisions are necessary. Moreover, combining cell-based functional data with genomic profiling may enable hybrid strategies that integrate both phenotypic and molecular markers of drug sensitivity [57].

In conclusion, cell line-based functional assays offer a practical, evidenced-supported approach to personalizing therapy in soft tissue sarcomas. While they do not replicate the complexity of in vivo models, their simplicity and demonstrated predictive value make them a valuable component of the functional precision oncology toolkit. Integrating these assays into clinical workflows, particularly in centers with translational oncology capabilities, may improve treatment selection and outcomes for patients with advanced or refractory STS [58,59]. 

### 3.2. Organoids

Organoids represent a cutting-edge functional assay platform that more closely mimics the three-dimensional structure and cellular complexity of original tumors compared to traditional two-dimensional cell cultures. Derived from patient tumor samples, organoids are miniature, self-organizing 3D cell cultures that recapitulate key histological and molecular features of the parental tumor, including its architecture, cell heterogeneity, elements of the tumor microenvironment [60].

**Figure 2:**
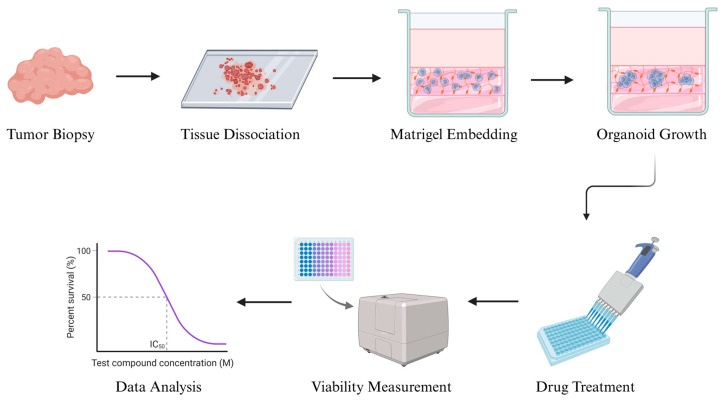
Stages of generation of soft-tissue sarcoma cell organoids and analysis of the efficacy of different drugs. Created in BioRender.com.

The concept of organoid culture was first developed in the early 2000s, initially in gastrointestinal and colorectal cancers, and later expanded to various solid tumors. The standard organoid culture process typically involves enzymatic and mechanical dissociation of fresh tumor tissue into small clusters or single cells, which are then embedded in an extracellular matrix scaffold such as Matrigel. The cells are grown in serum-free, growth factor-enriched media that support stem-like populations and promote tumor propagation. Over days to weeks, these cells proliferate and self-organize into 3D structures that preserve the original tumor’s histopathology and genomic features [61] (Figure 2).

In the context of soft tissue sarcomas (STSs), organoids offer a particularly promising platform due to the extreme histological diversity and intratumoral heterogeneity of these malignancies. Unlike traditional 2D cell line assays, organoids maintain spatial relationships between tumor cells and stromal components, as well as microenvironmental cues that may influence drug response [62]. As a result, 3D organoid models provide a more physiologically relevant system for evaluating therapeutic sensitivity and resistance compared to conventional cell line assays [63].

Rhabdomyosarcoma is one of the most extensively studied STS subtypes in organoid research. Organoids have been successfully generated from surgical specimens, biopsies, and even patient-derived xenografts, and can be maintained long-term while preserving the histological architecture, lineage-specific marker expression, and molecular features of the original tumor. These models yield sufficient viable cells for high-throughput drug screening and accurately reproduce known chemotherapy response patterns, with some studies identifying enhanced sensitivity to agents such as idasanutlin and navitoclax in functional assays [64]. Importantly, rhabdomyosarcoma organoids have also enabled mechanistic investigations; for example, CRISPR-based gene editing revealed that p53-deficient organoids exhibit increased susceptibility to checkpoint kinase inhibitors, highlighting subtype-specific therapeutic vulnerabilities that may not be evident through genomic profiling alone. Collectively, these findings demonstrate that rhabdomyosarcoma organoids are not only biologically faithful models but also clinically relevant tools for guiding personalized treatment strategies in high-risk pediatric sarcomas [65].

Beyond rhabdomyosarcoma, organoid technology has been successfully applied to a broad range of non-rhabdomyosarcoma STS subtypes. Organoids derived from myxoid liposarcoma and undifferentiated pleomorphic sarcoma preserve the morphological and genetic characteristics of the original tumors and have been used for high-throughput drug screening and phospho-proteomic profiling to identify activated signaling pathways and drug-response patterns [66]. Synovial sarcoma organoids retain the epigenetic consequences of SS18-SSX fusion and have enabled genome-wide chromatin analysis, revealing selective sensitivity to USP7 inhibition and uncovering novel subtype-specific vulnerabilities [67]. Fibrosarcoma organoids generated using 3D bioprinting platforms have facilitated direct comparison of tyrosine kinase inhibitors and anthracyclines, demonstrating distinct metabolic responses that correlate with clinical efficacy [68]. In addition, organoids and organoid-based transformation models have been used to study carcinosarcoma, where engineered alterations such as KRAS activation and TP53 or CDKN2A loss drive sarcomatous differentiation and reveal resistance to platinum-based chemotherapy but susceptibility to agents like paclitaxel and doxorubicin [69,70,71]. Collectively, these studies demonstrate that organoids can model the biological complexity of diverse STS subtypes and provide a functional platform for identifying subtype-specific therapeutic vulnerabilities.

Organoid models have also been successfully established from several other clinically important soft tissue sarcoma subtypes. Leiomyosarcoma organoids retain smooth muscle-specific markers and recurrent genomic alterations, enabling the evaluation of subtype-specific drug responses and targeted therapy strategies in tumors where actionable mutations are often limited. Dedifferentiated and pleomorphic liposarcoma organoids maintain their Large-scale sarcoma organoid biobanks have shown that patient-derived models can be reliably generated from multiple histological subtypes while preserving tumor heterogeneity and molecular features. High-throughput drug screening in these collections demonstrated strong concordance between organoid responses and clinical outcomes, supporting their value in predicting patient-specific therapy sensitivity. In addition, organoid-guided testing has identified effective treatments in cases where standard regimens failed, highlighting their potential role in personalized therapy for refractory STS. The ability to cryopreserve and longitudinally expand these models further enables adaptive treatment strategies as tumors evolve [72].

Sarcoma organoids have also been used to assess both chemotherapeutic and immunotherapeutic responses, maintaining the molecular and histopathological features of the original tumors while accurately reproducing known clinical sensitivities. Functional testing in these models has successfully identified alternative therapeutic options in patients who were resistant to standard regimens, demonstrating their value in guiding treatment selection. Moreover, immune-augmented and co-culture organoid systems have begun to reveal subtype-specific responses to checkpoint inhibition, suggesting a potential role for organoids in predicting immunotherapy benefit [73].

Although organoid culture systems offer significant advantages over traditional 2D assays, their implementation in STS remains challenging. Establishment success rates vary by subtype, protocols are not yet standardized across laboratories, and the generation of stable cultures can be time-consuming and technically demanding, which may limit their use in rapidly progressive disease. Furthermore, most current organoid models lack immune, stromal, and vascular components of the tumor microenvironment, reducing their ability to fully predict responses to immunotherapy or microenvironment-driven resistance. Despite these limitations, the ability to biobank and longitudinally expand organoids enables repeated drug testing over the course of treatment, supporting adaptive therapeutic strategies and real-time assessment of tumor evolution [74,75].

Overall, patient-derived organoids represent a major step forward in functional precision oncology for soft tissue sarcomas. By more faithfully recapitulating tumor biology than conventional models, they provide a versatile platform for drug screening, target discovery, and integration with genomic and transcriptomic data. While their routine clinical adoption will require further standardization and validation in prospective trials, organoid-based approaches hold substantial promise for enabling personalized treatment selection and improving outcomes in patients with these highly heterogeneous and therapeutically challenging tumors [76].

### 3.3. Xenografts

Patient-derived xenograft (PDX) models involve the transplantation of fresh human STS samples into immunodeficient mice, such as NOD/SCID or NSG strains, which lack adaptive immunity. This technique, first introduced in the 1960s and refined over subsequent decades, allows for the preservation of the tumor’s original architecture, cellular heterogeneity, and interactions with stromal components, features that are often lost in traditional in vitro systems [77,78].

The process typically begins with obtaining tumor tissue from surgical resections or core needle biopsies, which is then either enzymatically dissociated or directly implanted as small tissue fragments into recipient mice. Common implantation sites include subcutaneous tissue, orthotopic muscle locations, or the renal capsule, each offering varying degrees of physiological relevance to the tumor’s natural microenvironment. Following implantation, tumor growth is monitored over weeks to months, and engraftment success rates depend on factors such as STS subtype, tumor viability, and proliferative capacity [79,80].

**Figure 3:**
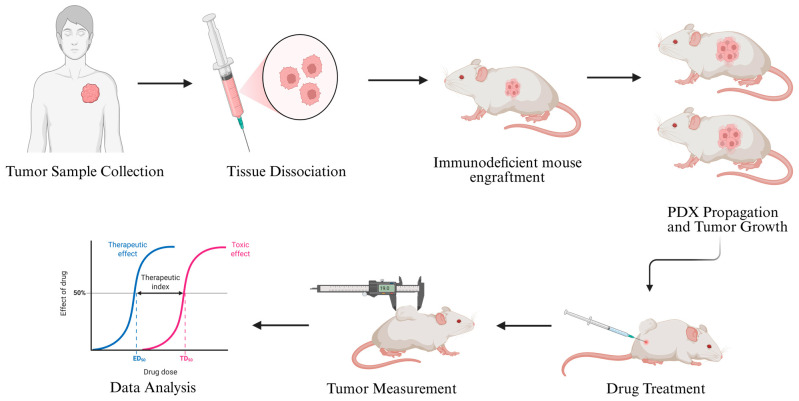
Generation of soft-tissue sarcoma patient-derived xenografts (PDX), implantation in mice and in vivo analysis of drug efficacy. Created in BioRender.com.

Once a PDX tumor is established, it can be serially passaged into additional mice, maintaining the histological, molecular, and phenotypic characteristics of the original patient tumor over multiple generations. This sustained propagation enables the development of PDX cohorts that can be used for functional drug testing. When tumors reach a predefined size, mice are randomized into control and treatment groups, and therapeutic agents—including chemotherapy, targeted therapies, or novel compounds—are administered according to dosing schedules designed to mimic clinical treatment protocols [81] (Figure 3).

Multiple studies have demonstrated that patient-derived xenograft models can be successfully established from a broad spectrum of soft tissue sarcoma subtypes, including leiomyosarcoma, undifferentiated pleomorphic sarcoma, synovial sarcoma, liposarcoma, and malignant peripheral nerve sheath tumor. These models retain the original tumor’s histopathological architecture, cellular heterogeneity, and immunophenotypic features, faithfully mirroring the morphology observed in patient samples. Molecular profiling—using techniques such as gene expression analysis, copy number variation assessment, and next-generation sequencing—has further confirmed that the genomic landscape of the original tumor, including characteristic mutations and structural alterations, remains preserved across multiple in vivo passages. This high degree of phenotypic and molecular stability establishes STS PDX models as reliable translational platforms, allowing long-term expansion, biobanking, and functional interrogation of tumor biology without loss of fidelity to the patient’s disease [79].

Patient-derived xenograft models have also been developed specifically from translocation-associated soft tissue sarcomas such as myxoid and round cell liposarcoma. These PDX models preserved hallmark molecular alterations, including the characteristic FUS-DDIT3 gene fusion, as well as the histologic transition between myxoid and round cell components, thereby accurately reflecting tumor progression and intratumoral heterogeneity. Importantly, these models were utilized for therapeutic evaluation and demonstrated sensitivity to agents known to be clinically effective in these subtypes, such as trabectedin, while reproducing patterns of resistance observed in patients. In addition to pharmacologic testing, these PDX systems enabled the exploration of subtype-specific biology, including tumor differentiation, signaling pathway activation, and microenvironmental interactions. By recapitulating both the molecular identity and drug response of the original tumors, these models provide a powerful platform for studying disease mechanisms and optimizing targeted treatment strategies in myxoid and round cell liposarcoma [82].

A recent multicenter clinical study provided one of the first real-world demonstrations of functional precision oncology in soft tissue sarcomas by using patient-derived xenograft (PDX) models to guide individualized treatment selection. In this study, tumor samples from patients with advanced or refractory STS were implanted into immunodeficient mice, expanded, and exposed to a panel of clinically relevant or investigational agents. Treatment recommendations were made based on in vivo drug response profiles and then applied to the corresponding patients when feasible. The PDX models successfully reproduced the histological and molecular features of the original tumors and generated drug sensitivity data within a timeframe compatible with clinical decision-making for a subset of patients. Importantly, several individuals who received PDX-guided therapies experienced meaningful clinical benefit, including partial responses and durable disease stabilization, particularly in translocation-associated and high-grade sarcoma subtypes. This study demonstrated the feasibility and potential clinical utility of PDX-guided personalized treatment in a prospective, multi-institutional setting. However, it also highlighted practical challenges, such as variable engraftment rates, long establishment times, and high resource requirements, indicating that while PDX-based strategies can inform therapy in selected patients, broader clinical integration will require streamlined workflows, enhanced scalability, and complementary rapid-turnaround functional platforms [82].

Moreover, PDX models have been instrumental in elucidating mechanisms of acquired drug resistance in STS. By performing integrative genomic and transcriptomic analyses on tumors before and after treatment, investigators have demonstrated dynamic clonal evolution and activation of compensatory signaling pathways under therapeutic pressure, highlighting the complexity of resistance beyond single-gene alterations [83]. In addition to monotherapy assessment, PDX-based studies have been used to test rational drug combinations in chemoresistant sarcoma models, identifying synergistic regimens that achieve greater tumor regression than single agents alone [84,85]. Orthotopic PDX models, in which tumor tissue is implanted into its native anatomical site, have also been developed to better preserve local microenvironmental interactions and metastatic behavior, thereby increasing the translational relevance of these systems for preclinical drug evaluation [86].

Nevertheless, PDX models remain one of the most physiologically relevant functional assays available in STS. Their ability to reproduce patient-specific tumor architecture, molecular profiles, and therapeutic responses in vivo provides unique insight into drug efficacy, resistance mechanisms, and tumor evolution. When integrated with genomic profiling, organoid systems, and other phenotypic assays, PDX models can serve as valuable tools for validating therapeutic strategies and informing personalized treatment development. However, given current logistical and biological constraints, their role is likely to remain complementary rather than standalone in clinical decision-making. In the future, the strategic combination of PDX with faster and more scalable functional platforms may improve feasibility and help translate preclinical findings into more individualized care, but robust prospective trials and standardized workflows will be essential before widespread clinical adoption can be achieved [87,88,89].

A table briefly summarizing the major advantages and limitations of the three major functional assay models is presented below (Table 3). 

## 4. Role of Tumor Microenvironment and Digital Spatial Profiling in Organoid and PDX Models

The tumor microenvironment (TME) plays a central role in the initiation, progression, immune evasion, and therapeutic resistance of solid malignancies, including soft tissue sarcomas (STSs) [90,91]. Unlike genetically uniform cell line systems, STS exhibit pronounced stromal content, variable immune infiltration, and significant spatial heterogeneity, all of which contribute to unpredictable treatment responses. The TME consists of cancer cells, fibroblasts, immune cells, endothelial cells, extracellular matrix (ECM), cytokines, and biomechanical forces that dynamically interact to shape tumor behavior [90]. Conventional two-dimensional cultures fail to reproduce these complex structural and functional interactions, limiting their relevance for predicting in vivo drug sensitivity [91].

Organoid models partially overcome these limitations by preserving three-dimensional architecture, cellular diversity, and ECM-dependent signaling [92]. Advanced organoid platforms incorporating co-culture systems, ECM scaffolds, or microfluidic “organ-on-a-chip” technologies have further enhanced the representation of fibroblasts, endothelial cells, and hypoxic gradients, allowing organoids to recapitulate aspects of the TME more accurately than 2D cultures [92,93]. Emerging studies have even explored microbiome–tumor interactions and metabolic crosstalk using next-generation organoid systems, highlighting their potential for modeling complex TME-driven resistance mechanisms [92]. However, organoids still face challenges in consistently maintaining immune components and full stromal architecture, which may limit their ability to evaluate immunotherapies or fully capture TME-mediated drug resistance.

Patient-derived xenograft (PDX) models provide an even more physiologically relevant representation of the TME by preserving native stromal elements, vascular architecture, and extracellular matrix organization within an in vivo setting [41,94]. Orthotopic implantation further recapitulates tissue-specific niches and metastatic behavior, allowing the study of invasion, angiogenesis, and metastatic dissemination. Recently, humanized PDX systems have reintroduced functional human immune cells, enabling investigation of tumor–immune interactions and response to checkpoint inhibitors in a clinically relevant context [94,95]. Despite these advantages, PDX models have important limitations: traditional models rely on immunodeficient mice, stromal replacement by murine cells can occur over serial passages, establishment times are long, success rates vary by subtype, and costs remain high [41,91,95]. These constraints limit their routine use in real-time clinical decision-making and highlight the need for complementary platforms.

Digital spatial profiling technologies have recently enabled high-resolution analysis of how drug responses vary across different regions of a tumor, uncovering spatial heterogeneity that cannot be captured by bulk assays or single-cell measurements. In patient-derived explant models, spatial immunostaining combined with computational analysis has demonstrated that distinct tumor regions exhibit variable sensitivity to the same agent, emphasizing the influence of local microenvironmental factors on treatment response [96]. Similarly, quantitative spatial analysis of metabolic activity across in vitro organoids and in vivo PDX models has revealed that organoids reproduce key molecular features but lack the spatial metabolic gradients and stromal complexity preserved in PDX, highlighting important differences in how each platform models the tumor microenvironment [97]. Together, these findings suggest that integrating functional assays such as organoids, PDX, and explants with spatial profiling approaches can provide a more comprehensive understanding of drug response in soft tissue sarcomas and help identify resistant niches that may inform biomarker development and personalized therapy.

Although the use of DSP in STS remains limited, integrating spatial profiling with functional platforms represents a promising future direction. Organoids offer scalable drug testing with partial TME fidelity, PDX models provide in vivo validation with high physiological relevance, and DSP enables high-resolution mapping of molecular and cellular interactions within these systems. Importantly, spatial analysis can reveal region-specific drug sensitivity and resistance patterns that are not detectable with bulk functional assays, making it particularly valuable for STS, which is defined by complex histology, stromal diversity, and heterogeneous treatment responses. Combining functional assays with spatially resolved TME analysis may therefore improve the prediction of therapeutic outcomes and facilitate the identification of novel biomarkers. However, prospective studies, standardized protocols, and multi-institutional validation are needed to determine how best to implement these integrated approaches in clinical practice.

## 5. Conventional Versus Personalized Therapy in Soft Tissue Sarcomas: Current Landscape and Future Directions

The management of STS has long relied on conventional oncology principles, in which treatment selection is primarily based on tumor histology, disease stage, and patient performance status. Standard first-line regimens, most commonly anthracycline chemotherapy with or without ifosfamide, have historically been applied across diverse patient populations, reflecting a uniform treatment approach. This strategy offers advantages such as broad availability, reproducibility, and support from decades of clinical trial data [98]. However, the biological diversity of STS subtypes means that many patients achieve only limited benefit, while others are exposed to significant toxicity without clear therapeutic gain. This disconnect highlights a major limitation of conventional therapy: it is designed around the “average” patient rather than the unique biology of each tumor [99].

Personalized medicine, or precision oncology, aims to address this limitation by aligning treatment decisions with the molecular and phenotypic features of individual tumors [86]. DNA-based assays, including targeted gene panels and sequencing technologies, have enabled biomarker-driven therapies in many malignancies such as lung cancer (EGFR inhibitors) and melanoma (BRAF inhibitors) [100]. In STS, however, actionable mutations are relatively rare, with the notable exception of KIT or PDGFRA alterations in gastrointestinal stromal tumors [101]. As a result, genomics alone often lacks predictive power in STS, prompting increasing interest in functional approaches that directly measure drug sensitivity using patient tumor material [102].

Functional precision oncology includes several complementary platforms. Two-dimensional cell line assays provide rapid and cost-effective drug sensitivity data and have shown meaningful concordance with clinical outcomes in STS. Three-dimensional organoids preserve tumor architecture and microenvironmental influences, offering a more physiologically relevant model and demonstrating strong alignment between ex vivo drug response and patient outcomes. Patient-derived xenograft (PDX) models remain the most faithful in vivo system, preserving tumor heterogeneity and stromal interactions; however, their long development times, high costs, and reliance on immunodeficient mice limit their feasibility for urgent clinical decision-making. Collectively, these functional platforms can enhance or even outperform genomics in predicting treatment response, particularly in genomic subtypes that lack clear actionable targets. 

Several distinctions emerge when comparing conventional and personalized strategies. Conventional therapy offers established safety profiles, lower initial costs, and wide applicability, yet lacks precision in biologically heterogeneous diseases and may result in overtreatment or ineffective care [103]. Personalized strategies—especially those combining molecular and functional data—provide greater accuracy in treatment selection, the potential to minimize toxicity, and the opportunity to identify novel therapeutic options. However, they also require specialized infrastructure, higher upfront resources, and may face logistical challenges in rapidly progressing disease [104]. Furthermore, many functional precision approaches in STS are currently supported by early-phase or retrospective studies, underscoring the need for prospective validation. 

A balanced path forward may lie in hybrid treatment algorithms. Rapid assays such as cell line-based platforms could be used early to guide timely decision-making, while organoids and PDX models could inform second-line or salvage regimens when more time is available. Genomic profiling would remain essential for diagnosis, subtype confirmation, and identification of rare actionable mutations, but functional validation could refine treatment selection, particularly in genomically silent tumors. Ultimately, integrating genomic, functional, and microenvironmental data within coordinated clinical workflows—and testing these strategies in prospective trials—may offer the most effective route toward truly personalized STS therapy [105].

## 6. Conclusions

Soft tissue sarcomas remain one of the most challenging malignancies in oncology due to their profound molecular heterogeneity, variable clinical behavior, and limited response to conventional therapies. While histology-driven treatment and anthracycline-based chemotherapy continue to serve as the standard of care, their modest efficacy underscores the limitations of uniform strategies in a biologically diverse disease. Advances in genomics have improved diagnostic accuracy and revealed occasional actionable mutations; however, genomics alone rarely predicts treatment response in most STS subtypes. 

Functional precision oncology has emerged as a complementary approach that directly measures drug sensitivity in patient-derived models, offering actionable insights beyond static molecular profiling. Cell line assays provide rapid screening, organoids recapitulate three-dimensional tumor architecture, and PDX models preserve in vivo complexity, each contributing unique strengths. More recently, digital spatial profiling has enabled characterization of the tumor microenvironment at high resolution, revealing immune and stromal factors that shape therapeutic outcomes. Together, these technologies point toward a future in which treatment is guided not by histology alone, but by an integrated understanding of tumor biology, phenotype, and spatial context. 

However, translation into routine clinical practice remains limited. Functional and spatial platforms require specialized infrastructure, access to high-quality tumor tissue, standardization of protocols, and reductions in cost and turnaround time. Most supporting evidence comes from early-phase or single-institution studies, highlighting the need for prospective, multi-center clinical trials that compare functional precision–guided therapy to current standards. Equitable access and healthcare policy support will also be essential to ensure that these advances benefit a broad patient population rather than select academic centers. 

In summary, the future management of STS will likely rely on hybrid treatment algorithms that combine histopathology, genomic profiling, functional drug testing, and spatial biology within coordinated clinical workflows. Such a multidimensional approach offers the potential to improve response rates, minimize toxicity, and identify novel therapeutic opportunities. However, significant challenges remain. The translational gap is still obvious, and there is an obvious need for practice harmonization between different centers so that the results of functional assays can be widely applicable. Furthermore, there are significant regulatory and reimbursement barriers to implementing hybrid models that unify genomic, transcriptomic, and functional data, thereby advancing the treatment of STS. 

## Figures and Tables

**Table 1:** Main chemotherapeutic regimens for STS.

Drug	Indication/Sarcoma Type	Response Rate (%)	Median OS (Months)
Doxorubicin	Standard first-line for most advanced STS types	16.0–27.0	7.7–12.8
Doxorubicin + Ifosfamide	High-risk patients, younger age, combination therapy	14.0–34.0	7.3–14.3
Gemcitabine + Docetaxel	Leiomyosarcoma, UPS, especially uterine LMS	5.0–52.0	16.0–26.9
Pazopanib	Non-adipocytic STS (leiomyosarcoma, synovial sarcoma)	6.0	12.5
Eribulin	Liposarcoma, leiomyosarcoma	4.0	13.6
Ifosfamide	Various STS types, especially synovial sarcoma	20.0–25.0	12.0
Gemcitabine (single agent)	Various STS types	3.2–27.0	7.2–20.0
Dacarbazine	Various STS types	4.0–18.0	8.2–11.5

**Table 2:** Commonly used soft tissue sarcoma cell lines in functional drug sensitivity studies.

Cell Line	STS Subtype	Origin/Key Features	Functional Applications	Known Drug Sensitivity/Use
*HT-1080*	Fibrosarcoma/UPS model	Metastatic fibrosarcoma; high proliferation	2D assays, invasion, pathway studies	Sensitive to doxorubicin, ifosfamide; used in novel drug screens
*NMFH-1*	Undifferentiated Pleomorphic Sarcoma	Derived from MFH; high-grade	Drug screening, apoptosis assays	Variable sensitivity to anthracyclines/TKIs
*FU-MFH-13*	Undifferentiated Pleomorphic Sarcoma	Recurrent MFH tumor	Chemoresistance and phenotypic assays	Models high-grade UPS resistance
*SK-LMS-1*	Leiomyosarcoma	Uterine LMS; SMA positive	Cytotoxic testing, hormone studies	Moderate response to trabectedin, pazopanib
*SK-UT-1/SK-UT-1B*	Leiomyosarcoma	Uterine LMS; widely used model	2D assays, gene expression studies	Variable doxorubicin/ifosfamide response
*SYO-1*	Synovial Sarcoma	SS18-SSX fusion positive	Targeted therapy, epigenetic inhibitor studies	Sensitive to HDAC/EZH2 inhibitors
*HS-SY-II*	Synovial Sarcoma	SS18-SSX2 fusion	Functional and invasion assays	Tested with multi-kinase and epigenetic agents
*SW872*	Well-diff Liposarcoma	Primary liposarcoma; moderate growth	Drug screening, adipogenic studies	Variable response to gemcitabine, pazopanib
*402–91*	Myxoid/Round cell Liposarcoma	FUS-DDIT3 fusion positive	Fusion-targeted drug testing	Sensitive to trabectedin
*STS26T*	MPNST	Recurrent MPNST; aggressive	Targeted drug screening, MEK/PI3K studies	Sensitive to MEK inhibitors

**Table 3:** Overview of advantages and limitations of the major functional assays in soft tissue sarcomas.

Functional Assay Type	Physiological Relevance	Advantages	Limitations
Cell lines	Low	Relatively simple, shorter processing time	Limited predictive precision, possible selection bias, no representation of the tumor microenvironment
Organoids	Average	Match original tumor histology and microenvironment to a satisfactory degree, no requirement for laboratory animals	Technical complexity, long processing time. incomplete tumor microenvironment representation
Xenografts	High	More accurate representation of tumor architecture and microenvironment	High cost, technical complexity, long processing time. ethical considerations, limited scalability

## Data Availability

No new data were created.

## Abbreviations

The following abbreviations are used in this manuscript:

STS	Soft-Tissue Sarcoma
MTT	Dimethylthiazol Diphenyltetrazolium Bromide
PDX	Patient-Derived Xenografts
ATP	Adenosine Triphosphate
EGFR	Epidermal Growth Factor Receptor
PCR	Polymerase Chain Reaction
NOD/SCID	Nonobese diabetic/severe combined immunodeficiency
PDGFR	Platelet-derived Growth Factor Receptor

## References

[1] Hui J.Y. (2016). Epidemiology and Etiology of Sarcomas. Surg. Clin. N. Am..

[2] Sbaraglia M., Dei Tos A.P. (2019). The pathology of soft tissue sarcomas. Radiol. Med..

[3] Gronchi A., Miah A.B., Dei Tos A.P., Abecassis N., Bajpai J., Bauer S., Biagini R., Bielack S., Blay J.Y., Bolle S. (2021). Soft tissue and visceral sarcomas: ESMO-EURACAN-GENTURIS Clinical Practice Guidelines for diagnosis, treatment and follow-up☆. Ann. Oncol..

[4] Lewis J.J., Brennan M.F. (1996). Soft tissue sarcomas. Curr. Probl. Surg..

[5] Spiker A.M., Mangla A., Ramsey M.L. (2025). Angiosarcoma.

[6] Gilbert N.F., Cannon C.P., Lin P.P., Lewis V.O. (2009). Soft-tissue sarcoma. J. Am. Acad. Orthop. Surg..

[7] Harris S.J., Benson C., Jones R.L. (2015). Current and advancing systemic treatment options for soft tissue sarcomas. Expert Opin. Pharmacother..

[8] Spolverato G., Callegaro D., Gronchi A. (2020). Defining Which Patients Are at High Risk for Recurrence of Soft Tissue Sarcoma. Curr. Treat. Options Oncol..

[9] Colombo C., Randall R.L., Andtbacka R.H., Gronchi A. (2012). Surgery in soft tissue sarcoma: More conservative in extremities, more extended in the retroperitoneum. Expert Rev. Anticancer Ther..

[10] Gronchi A., Maki R.G., Jones R.L. (2017). Treatment of soft tissue sarcoma: A focus on earlier stages. Future Oncol..

[11] Correa R., Gómez-Millán J., Lobato M., Fernández A., Ordoñez R., Castro C., Lupiañez Y., Medina J.A. (2018). Radiotherapy in soft-tissue sarcoma of the extremities. Clin. Transl. Oncol..

[12] Hewitt L., Powell R., Zenginer K., Coyle C., Murray H., Cooper L., Gregory J. (2019). Patient Perceptions of the Impact of Treatment (Surgery and Radiotherapy) for Soft Tissue Sarcoma. Sarcoma.

[13] Salerno K.E., Alektiar K.M., Baldini E.H., Bedi M., Bishop A.J., Bradfield L., Chung P., DeLaney T.F., Folpe A., Kane J.M. (2021). Radiation Therapy for Treatment of Soft Tissue Sarcoma in Adults: Executive Summary of an ASTRO Clinical Practice Guideline. Pract. Radiat. Oncol..

[14] Eilber F.R., Huth J.F., Mirra J., Rosen G. (1990). Progress in the recognition and treatment of soft tissue sarcomas. Cancer.

[15] Neuville A., Chibon F., Coindre J.M. (2014). Grading of soft tissue sarcomas: From histological to molecular assessment. Pathology.

[16] Gamboa A.C., Gronchi A., Cardona K. (2020). Soft-tissue sarcoma in adults: An update on the current state of histiotype-specific management in an era of personalized medicine. CA Cancer J. Clin..

[17] Grünewald T.G., Alonso M., Avnet S., Banito A., Burdach S., Cidre-Aranaz F., Di Pompo G., Distel M., Dorado-Garcia H., Garcia-Castro J. (2020). Sarcoma treatment in the era of molecular medicine. EMBO Mol. Med..

[18] Liebner D.A. (2015). The indications and efficacy of conventional chemotherapy in primary and recurrent sarcoma. J. Surg. Oncol..

[19] Ratan R., Patel S.R. (2016). Chemotherapy for soft tissue sarcoma. Cancer.

[20] Judson I., Verweij J., Gelderblom H., Hartmann J.T., Schöffski P., Blay J.Y., Kerst J.M., Sufliarsky J., Whelan J., Hohenberger P. (2014). Doxorubicin alone versus intensified doxorubicin plus ifosfamide for first-line treatment of advanced or metastatic soft-tissue sarcoma: A randomised controlled phase 3 trial. Lancet Oncol..

[21] Woll P.J., Reichardt P., Le Cesne A., Bonvalot S., Azzarelli A., Hoekstra H.J., Leahy M., Van Coevorden F., Verweij J., Hogendoorn P.C. (2012). Adjuvant chemotherapy with doxorubicin, ifosfamide, and lenograstim for resected soft-tissue sarcoma (EORTC 62931): A multicentre randomised controlled trial. Lancet Oncol..

[22] Pervaiz N., Colterjohn N., Farrokhyar F., Tozer R., Figueredo A., Ghert M. (2008). A systematic meta-analysis of randomized controlled trials of adjuvant chemotherapy for localized resectable soft-tissue sarcoma. Cancer.

[23] Grignani G., Le Cesne A., Martín-Broto J. (2022). Trabectedin as second-line treatment in advanced soft tissue sarcoma: Quality of life and safety outcomes. Future Oncol..

[24] Le Cesne A., Martín-Broto J., Grignani G. (2022). A review of the efficacy of trabectedin as second-line treatment of advanced soft tissue sarcoma. Future Oncol..

[25] Skafida E., Kokkali S., Nikolaou M., Digklia A. (2017). Metastatic soft tissue sarcoma: Current treatment landscape and future perspectives. Expert Rev. Anticancer Ther..

[26] Naito Y., Aburatani H., Amano T., Baba E., Furukawa T., Hayashida T., Hiyama E., Ikeda S., Kanai M., Kato M. (2021). Clinical practice guidance for next-generation sequencing in cancer diagnosis and treatment (edition 2.1). Int. J. Clin. Oncol..

[27] Casolino R., Beer P.A., Chakravarty D., Davis M.B., Malapelle U., Mazzarella L., Normanno N., Pauli C., Subbiah V., Turnbull C. (2024). Interpreting and integrating genomic tests results in clinical cancer care: Overview and practical guidance. CA Cancer J. Clin..

[28] Pascual J., Attard G., Bidard F.C., Curigliano G., De Mattos-Arruda L., Diehn M., Italiano A., Lindberg J., Merker J.D., Montagut C. (2022). ESMO recommendations on the use of circulating tumour DNA assays for patients with cancer: A report from the ESMO Precision Medicine Working Group. Ann. Oncol..

[29] Leal A., Sidransky D., Brait M. (2020). Tissue and Cell-Free DNA-Based Epigenomic Approaches for Cancer Detection. Clin. Chem..

[30] Volckmar A.L., Sültmann H., Riediger A., Fioretos T., Schirmacher P., Endris V., Stenzinger A., Dietz S. (2018). A field guide for cancer diagnostics using cell-free DNA: From principles to practice and clinical applications. Genes Chromosomes Cancer.

[31] Zhang K., Fu R., Liu R., Su Z. (2024). Circulating cell-free DNA-based multi-cancer early detection. Trends Cancer.

[32] Song P., Wu L.R., Yan Y.H., Zhang J.X., Chu T., Kwong L.N., Patel A.A., Zhang D.Y. (2022). Limitations and opportunities of technologies for the analysis of cell-free DNA in cancer diagnostics. Nat. Biomed. Eng..

[33] Jain S., Xu R., Prieto V.G., Lee P. (2010). Molecular classification of soft tissue sarcomas and its clinical applications. Int. J. Clin. Exp. Pathol..

[34] Morand du Puch C.B., Vanderstraete M., Giraud S., Lautrette C., Christou N., Mathonnet M. (2021). Benefits of functional assays in personalized cancer medicine: More than just a proof-of-concept. Theranostics.

[35] Letai A., Bhola P., Welm A.L. (2022). Functional precision oncology: Testing tumors with drugs to identify vulnerabilities and novel combinations. Cancer Cell.

[36] Stoddart M.J. (2011). Cell viability assays: Introduction. Methods Mol. Biol..

[37] Drost J., Clevers H. (2018). Organoids in cancer research. Nat. Rev. Cancer.

[38] Fang Z., Li P., Du F., Shang L., Li L. (2023). The role of organoids in cancer research. Exp. Hematol. Oncol..

[39] LeSavage B.L., Suhar R.A., Broguiere N., Lutolf M.P., Heilshorn S.C. (2022). Next-generation cancer organoids. Nat. Mater..

[40] Liu Y., Wu W., Cai C., Zhang H., Shen H., Han Y. (2023). Patient-derived xenograft models in cancer therapy: Technologies and applications. Signal Transduct. Target. Ther..

[41] Jung J., Seol H.S., Chang S. (2018). The Generation and Application of Patient-Derived Xenograft Model for Cancer Research. Cancer Res. Treat..

[42] Ciraku L., Moeller R.A., Esquea E.M., Gocal W.A., Hartsough E.J., Simone N.L., Jackson J.G., Reginato M.J. (2021). An Ex Vivo Brain Slice Model to Study and Target Breast Cancer Brain Metastatic Tumor Growth. J. Vis. Exp..

[43] Martin S.Z., Wagner D.C., Hörner N., Horst D., Lang H., Tagscherer K.E., Roth W. (2019). Ex vivo tissue slice culture system to measure drug-response rates of hepatic metastatic colorectal cancer. BMC Cancer.

[44] Roelants C., Pillet C., Franquet Q., Sarrazin C., Peilleron N., Giacosa S., Guyon L., Fontanell A., Fiard G., Long J.A. (2020). Ex-Vivo Treatment of Tumor Tissue Slices as a Predictive Preclinical Method to Evaluate Targeted Therapies for Patients with Renal Carcinoma. Cancers.

[45] Jung S., Jo H., Hyung S., Jeon N.L. (2022). Advances in 3D Vascularized Tumor-on-a-Chip Technology. Adv. Exp. Med. Biol..

[46] Ayuso J.M., Rehman S., Farooqui M., Virumbrales-Muñoz M., Setaluri V., Skala M.C., Beebe D.J. (2020). Microfluidic Tumor-on-a-Chip Model to Study Tumor Metabolic Vulnerability. Int. J. Mol. Sci..

[47] Chakrabarty S., Quiros-Solano W.F., Kuijten M.M.P., Haspels B., Mallya S., Lo C.S.Y., Othman A., Silvestri C., van de Stolpe A., Gaio N. (2022). A Microfluidic Cancer-on-Chip Platform Predicts Drug Response Using Organotypic Tumor Slice Culture. Cancer Res..

[48] Esner M., Meyenhofer F., Bickle M. (2018). Live-Cell High Content Screening in Drug Development. Methods Mol. Biol..

[49] Nierode G., Kwon P.S., Dordick J.S., Kwon S.J. (2016). Cell-Based Assay Design for High-Content Screening of Drug Candidates. J. Microbiol. Biotechnol..

[50] Kumar P., Nagarajan A., Uchil P.D. (2018). Analysis of Cell Viability by the MTT Assay. Cold Spring Harb. Protoc..

[51] Adan A., Kiraz Y., Baran Y. (2016). Cell Proliferation and Cytotoxicity Assays. Curr. Pharm. Biotechnol..

[52] Kamiloglu S., Sari G., Ozdal T., Capanoglu E. (2020). Guidelines for cell viability assays. Food Front..

[53] Lomakina G.Y., Modestova Y.A., Ugarova N.N. (2015). Bioluminescence assay for cell viability. Biochemistry.

[54] Sánchez-Díez M., Romero-Jiménez P., Alegría-Aravena N., Gavira-O’Neill C.E., Vicente-García E., Quiroz-Troncoso J., González-Martos R., Ramírez-Castillejo C., Pastor J.M. (2025). Assessment of Cell Viability in Drug Therapy: IC50 and Other New Time-Independent Indices for Evaluating Chemotherapy Efficacy. Pharmaceutics.

[55] Fetisov T.I., Khazanova S.A., Shtompel P.A., Trapeznikova E.S., Zinovieva V.Y., Marshall V.I., Lovenger A.A., Rogozhin D.V., Anastasia T.A., Bokhyan B.Y. (2023). Perspectives of Cell Sensitivity/Resistance Assay in Soft Tissue Sarcomas Chemotherapy. Int. J. Mol. Sci..

[56] Ball C.R., Fröhling S. (2024). Let’s get functional: Drug sensitivity profiling to enable precision sarcoma medicine. Cell Stem Cell.

[57] Gao T., He X., Wang J., Liu J., Hu X., Bai C., Yin S., Shi Y., Wang Y., Tan Z. (2025). Self-assembled patient-derived tumor-like cell clusters for personalized drug testing in diverse sarcomas. Cell Rep. Med..

[58] De Vita A., Mercatali L., Miserocchi G., Liverani C., Spadazzi C., Recine F., Bongiovanni A., Pieri F., Cavaliere D., Fausti V. (2018). Establishment of a Primary Culture of Patient-derived Soft Tissue Sarcoma. J. Vis. Exp..

[59] Subbiah V. (2014). Prospects and pitfalls of personalizing therapies for sarcomas: From children, adolescents, and young adults to the elderly. Curr. Oncol. Rep..

[60] Zhao Z., Chen X., Dowbaj A.M., Sljukic A., Bratlie K., Lin L., Fong E.L.S., Balachander G.M., Chen Z., Soragni A. (2022). Organoids. Nat. Rev. Methods Primers.

[61] Corrò C., Novellasdemunt L., Li V.S.W. (2020). A brief history of organoids. Am. J. Physiol. Cell Physiol..

[62] Psilopatis I., Kokkali S., Palamaris K., Digklia A., Vrettou K., Theocharis S. (2022). Organoids: A New Chapter in Sarcoma Diagnosis and Treatment. Int. J. Mol. Sci..

[63] Colella G., Fazioli F., Gallo M., De Chiara A., Apice G., Ruosi C., Cimmino A., de Nigris F. (2018). Sarcoma Spheroids and Organoids-Promising Tools in the Era of Personalized Medicine. Int. J. Mol. Sci..

[64] Gatzweiler C., Ridinger J., Herter S., Gerloff X.F., ElHarouni D., Berker Y., Imle R., Schmitt L., Kreth S., Stainczyk S. (2022). Functional Therapeutic Target Validation Using Pediatric Zebrafish Xenograft Models. Cancers.

[65] Meister M.T., Groot Koerkamp M.J.A., de Souza T., Breunis W.B., Frazer-Mendelewska E., Brok M., DeMartino J., Manders F., Calandrini C., Kerstens H.H.D. (2022). Mesenchymal tumor organoid models recapitulate rhabdomyosarcoma subtypes. EMBO Mol. Med..

[66] Gaebler M., Silvestri A., Reichardt P., Wardelmann E., Gambara G., Haybaeck J., Stroebel P., Niethard M., Erdmann G., Regenbrecht C.R. (2019). Abstract 469: Patient-derived sarcoma models: First results from the SARQMA study. Cancer Res..

[67] Boulay G., Cironi L., Garcia S.P., Rengarajan S., Xing Y.-H., Lee L., Awad M.E., Naigles B., Iyer S., Broye L.C. (2021). The chromatin landscape of primary synovial sarcoma organoids is linked to specific epigenetic mechanisms and dependencies. Life Sci. Alliance.

[68] Maloney E., Clark C., Sivakumar H., Yoo K., Aleman J., Rajan S.A.P., Forsythe S., Mazzocchi A., Laxton A.W., Tatter S.B. (2020). Immersion Bioprinting of Tumor Organoids in Multi-Well Plates for Increasing Chemotherapy Screening Throughput. Micromachines.

[69] Maru Y., Tanaka N., Tatsumi Y., Nakamura Y., Itami M., Hippo Y. (2021). Kras activation in endometrial organoids drives cellular transformation and epithelial-mesenchymal transition. Oncogenesis.

[70] Maru Y., Tanaka N., Tatsumi Y., Nakamura Y., Yao R., Noda T., Itami M., Hippo Y. (2021). Probing the tumorigenic potential of genetic interactions reconstituted in murine fallopian tube organoids. J. Pathol..

[71] McCorkle J.R., Burgess B.T., McDowell A.B., DeJohn J., DeSimone C.P., Ueland F.R., Kolesar J.M., Gorski J.W. (2020). Abstract B22: Development of the first ovarian carcinosarcoma patient-derived xenograft and tissue organoid model to predict clinical response to chemotherapy. Clin. Cancer Res..

[72] Al Shihabi A., Tebon P.J., Nguyen H.T.L., Chantharasamee J., Sartini S., Davarifar A., Jensen A.Y., Diaz-Infante M., Cox H., Gonzalez A.E. (2024). The landscape of drug sensitivity and resistance in sarcoma. Cell Stem Cell.

[73] Ma H., Li X., Li J., Bu J., Li X., Zhang J., Yu S., Nie G., Wang H., Feng H. (2026). Generation of patient-derived sarcoma organoids for personalized drug screening and precision cancer immunotherapy. Biomaterials.

[74] Li Y., Tang P., Cai S., Peng J., Hua G. (2020). Organoid based personalized medicine: From bench to bedside. Cell Regen..

[75] Zhou Z., Cong L., Cong X. (2021). Patient-Derived Organoids in Precision Medicine: Drug Screening, Organoid-on-a-Chip and Living Organoid Biobank. Front. Oncol..

[76] Xu S., Tan S., Guo L. (2023). Patient-Derived Organoids as a Promising Tool for Multimodal Management of Sarcomas. Cancers.

[77] Hidalgo M., Amant F., Biankin A.V., Budinská E., Byrne A.T., Caldas C., Clarke R.B., de Jong S., Jonkers J., Mælandsmo G.M. (2014). Patient-derived xenograft models: An emerging platform for translational cancer research. Cancer Discov..

[78] Gu A., Li J., Li M.-Y., Liu Y. (2025). Patient-derived xenograft model in cancer: Establishment and applications. MedComm.

[79] Cornillie J., Wozniak A., Li H., Wang Y., Boeckx B., Gebreyohannes Y.K., Wellens J., Vanleeuw U., Hompes D., Stas M. (2019). Establishment and Characterization of Histologically and Molecularly Stable Soft-tissue Sarcoma Xenograft Models for Biological Studies and Preclinical Drug Testing. Mol. Cancer Ther..

[80] Lu W., Chao T., Ruiqi C., Juan S., Zhihong L. (2018). Patient-derived xenograft models in musculoskeletal malignancies. J. Transl. Med..

[81] Russell T.A., Eckardt M.A., Murakami T., Elliott I.A., Kawaguchi K., Kiyuna T., Igarashi K., Li Y., Crompton J.G., Graham D.S. (2017). Clinical Factors That Affect the Establishment of Soft Tissue Sarcoma Patient-Derived Orthotopic Xenografts: A University of California, Los Angeles, Sarcoma Program Prospective Clinical Trial. JCO Precis. Oncol..

[82] Qi Y., Hu Y., Yang H., Zhuang R., Hou Y., Tong H., Feng Y., Huang Y., Jiang Q., Ji Q. (2017). Establishing a patient-derived xenograft model of human myxoid and round-cell liposarcoma. Oncotarget.

[83] Aoki Y., Yamamoto J., Tome Y., Hamada K., Masaki N., Inubushi S., Tashiro Y., Bouvet M., Endo I., Nishida K. (2021). Over-methylation of Histone H3 Lysines Is a Common Molecular Change Among the Three Major Types of Soft-tissue Sarcoma in Patient-derived Xenograft (PDX) Mouse Models. Cancer Genom.-Proteom..

[84] Higuchi T., Igarashi K., Yamamoto N., Hayashi K., Kimura H., Miwa S., Bouvet M., Tsuchiya H., Hoffman R.M. (2022). Review: Precise sarcoma patient-derived orthotopic xenograft (PDOX) mouse models enable identification of novel effective combination therapies with the cyclin-dependent kinase inhibitor palbociclib: A strategy for clinical application. Front. Oncol..

[85] Tseng J.-R., Hsu C.-L., Hsieh H.-H., Ho K.-C., Chung Y.-H., Wu C.-Y. (2024). The synergy of 177Lu-FAPI-46 with tyrosine kinase inhibitor in a sarcoma patient-derived xenograft mouse model. Biomed. J..

[86] Igarashi K., Kawaguchi K., Murakami T., Miyake K., Kiyuna T., Miyake M., Hiroshima Y., Higuchi T., Oshiro H., Nelson S.D. (2020). Patient-derived orthotopic xenograft models of sarcoma. Cancer Lett..

[87] Abdolahi S., Ghazvinian Z., Muhammadnejad S., Saleh M., Asadzadeh Aghdaei H., Baghaei K. (2022). Patient-derived xenograft (PDX) models, applications and challenges in cancer research. J. Transl. Med..

[88] Jin J., Yoshimura K., Sewastjanow-Silva M., Song S., Ajani J.A. (2023). Challenges and Prospects of Patient-Derived Xenografts for Cancer Research. Cancers.

[89] Stebbing J., Paz K., Schwartz G.K., Wexler L.H., Maki R., Pollock R.E., Morris R., Cohen R., Shankar A., Blackman G. (2014). Patient-derived xenografts for individualized care in advanced sarcoma. Cancer.

[90] Giraldo N.A., Sanchez-Salas R., Peske J.D., Vano Y., Becht E., Petitprez F., Validire P., Ingels A., Cathelineau X., Fridman W.H. (2019). The clinical role of the TME in solid cancer. Br. J. Cancer.

[91] Sun Y. (2016). Tumor microenvironment and cancer therapy resistance. Cancer Lett..

[92] Xia T., Du W.-L., Chen X.-Y., Zhang Y.-N. (2021). Organoid models of the tumor microenvironment and their applications. J. Cell. Mol. Med..

[93] Devarasetty M., Forsythe S.D., Shelkey E., Soker S. (2020). In Vitro Modeling of the Tumor Microenvironment in Tumor Organoids. Tissue Eng. Regen. Med..

[94] Pu X., Zhang R., Wang L., Chen Y., Xu Y., Pataer A., Meraz I.M., Zhang X., Wu S., Wu L. (2018). Patient-derived tumor immune microenvironments in patient-derived xenografts of lung cancer. J. Transl. Med..

[95] Zhao Y., Shuen T.W.H., Toh T.B., Chan X.Y., Liu M., Tan S.Y., Fan Y., Yang H., Lyer S.G., Bonney G.K. (2018). Development of a new patient-derived xenograft humanised mouse model to study human-specific tumour microenvironment and immunotherapy. Gut.

[96] Miles G.J., Powley I., Mohammed S., Howells L., Pringle J.H., Hammonds T., MacFarlane M., Pritchard C. (2021). Evaluating and comparing immunostaining and computational methods for spatial profiling of drug response in patient-derived explants. Lab. Investig..

[97] Heaster T.M., Landman B.A., Skala M.C. (2019). Quantitative Spatial Analysis of Metabolic Heterogeneity Across In Vivo and In Vitro Tumor Models. Front. Oncol..

[98] Grimer R., Judson I., Peake D., Seddon B. (2010). Guidelines for the management of soft tissue sarcomas. Sarcoma.

[99] Badalamenti G., Messina C., De Luca I., Musso E., Casarin A., Incorvaia L. (2019). Soft tissue sarcomas in the precision medicine era: New advances in clinical practice and future perspectives. Radiol. Medica.

[100] Damodaran S., Berger M.F., Roychowdhury S. (2015). Clinical tumor sequencing: Opportunities and challenges for precision cancer medicine. Am. Soc. Clin. Oncol. Educ. Book.

[101] Demetri G.D., Mehren M.V., Blanke C.D., Abbeele A.D.V.d., Eisenberg B., Roberts P.J., Heinrich M.C., Tuveson D.A., Singer S., Janicek M. (2002). Efficacy and Safety of Imatinib Mesylate in Advanced Gastrointestinal Stromal Tumors. N. Engl. J. Med..

[102] van Renterghem A.W.J., van de Haar J., Voest E.E. (2023). Functional precision oncology using patient-derived assays: Bridging genotype and phenotype. Nat. Rev. Clin. Oncol..

[103] Tannock I.F. (1998). Conventional cancer therapy: Promise broken or promise delayed?. Lancet.

[104] Wang R.C., Wang Z. (2023). Precision Medicine: Disease Subtyping and Tailored Treatment. Cancers.

[105] Chan S.P.Y., Rashid M.B.M.A., Lim J.J., Goh J.J.N., Wong W.Y., Hooi L., Ismail N.N., Luo B., Chen B.J., Noor N.F.B.M. (2025). Functional combinatorial precision medicine for predicting and optimizing soft tissue sarcoma treatments. NPJ Precis. Oncol..

